# Identification of miRNA Signature Associated With Erectile Dysfunction in Type 2 Diabetes Mellitus by Support Vector Machine-Recursive Feature Elimination

**DOI:** 10.3389/fgene.2021.762136

**Published:** 2021-10-11

**Authors:** Haibo Xu, Baoyin Zhao, Wei Zhong, Peng Teng, Hong Qiao

**Affiliations:** ^1^ The Second Affiliated Hospital of Harbin Medical University, Harbin, China; ^2^ The First Hospital of Qiqihar, Qiqihar, China

**Keywords:** micrornas, diabetes mellitus, erectile dysfunction, signature, molecular mechanisms

## Abstract

Diabetic mellitus erectile dysfunction (DMED) is one of the most common complications of diabetes mellitus (DM), which seriously affects the self-esteem and quality of life of diabetics. MicroRNAs (miRNAs) are endogenous non-coding RNAs whose expression levels can affect multiple cellular processes. Many pieces of studies have demonstrated that miRNA plays a role in the occurrence and development of DMED. However, the exact mechanism of this process is unclear. Hence, we apply miRNA sequencing from blood samples of 10 DMED patients and 10 DM controls to study the mechanisms of miRNA interactions in DMED patients. Firstly, we found four characteristic miRNAs as signature by the SVM-RFE method (hsa-let-7E-5p, hsa-miR-30 days-5p, hsa-miR-199b-5p, and hsa-miR-342–3p), called DMEDSig-4. Subsequently, we correlated DMEDSig-4 with clinical factors and further verified the ability of these miRNAs to classify samples. Finally, we functionally verified the relationship between DMEDSig-4 and DMED by pathway enrichment analysis of miRNA and its target genes. In brief, our study found four key miRNAs, which may be the key influencing factors of DMED. Meanwhile, the DMEDSig-4 could help in the development of new therapies for DMED.

## Introduction

Erectile dysfunction (ED) refers to the persistent or repeated failure of men to achieve and/or maintain penile erection for satisfactory sexual activity. As a common and the most neglected complication of diabetes (Zhao et al., 2020; Long et al., 2021; Yang et al., 2021), diabetic mellitus erectile dysfunction (DMED) is an important factor affecting psychological well-being, spousal relationship and family life (Malavige and Levy, 2009). The massive research indicated patient of T2MD incidence ED was significantly higher than that of the health. 75%male with diabetes is affected with ED. 66.3% is T2MD among of the data (Kouidrat et al., 2017; Cheng et al., 2018; Zagidullin et al., 2019; Zhu et al., 2021a). DMED is considered as an alternative marker for diabetes and cardiovascular disease, and is the primary feature of diabetes. Meanwhile, DMED has a multifactorial pathological process that can occur simultaneously with cardiovascular disease, neuropathy, and depression. How to effectively intervene in DMED has become an urgent problem in the global medical community.

MicroRNAs (miRNAs) are small non-coding RNAs of 19–25 nucleotides (Cheng et al., 2016; Lu and Rothenberg, 2018; Wu et al., 2019; Mo et al., 2020). They have been used as important regulators of gene expression in recent decades. Changes in their expression levels can affect multiple cellular processes and are used as molecular markers for diagnosis and follow-up (Han et al., 2021). It is widely involved in pathological processes such as cancer (Rupaimoole and Slack, 2017; Liu et al., 2021a; Lei and Shu-Lin, 2021; Sheng et al., 2021; Tang et al., 2021), DM (Vasu et al., 2019), cardiovascular events (Barwari et al., 2016), and ED (Ding et al., 2017). However, there are few related studies in DMED.

Growing evidences have indicated that miRNAs play an important role in the occurrence and development of DM and diabetic complications (Kong et al., 2011; Jiang et al., 2015). Unfortunately, the exact pathogenesis of miRNAs action on DMED remains to be largely unknown. Hence, we adopted the machine learning method (SVM-RFE), identified the characteristic miRNAs of DMED, constructed the signature of DMED and found potentially related pathways. Our work has significance for the identification of the molecular mechanism and the early prediction and diagnosis of DMED.

## Materials and Methods

### Subjects

Inclusion criteria: T2DM patients admitted to Qiqihar Medical College from December 2020 to June 2021 were selected as the study subjects. Erectile dysfunction was identified by international index of erectile function -5 (IIEF-5). Diagnostic criteria for T2DM: in line with WHO diabetes diagnostic criteria in 1999; Diagnostic and grading criteria for ED: 1) Regular sexual partner and normal sexual life; 2) History of ED for more than 6 months; 3) Erectile dysfunction was assessed according to IIEF-5, and the total score ≤21 was divided into ED; A score of five to seven was severe, 8–11 was moderate, 12–21 was mild, and ≥22 was no ED. Healthy married men with erectile dysfunction aged 30–70 years with a course of 2–10 years were included in the study.

Exclusion criteria: 1) The informed consent is not signed or the medical records are incomplete; 2) Incomplete research data; 3) Type 1 diabetes mellitus (T1DM), adult latent autoimmune diabetes mellitus (LADA), acute complications of diabetes mellitus; 4) Hypogonadism, thyroid disease, adrenal disease, pituitary disease, etc.; 5) Pelvic and urinary genital malformations, inflammation, tumor, trauma, surgical history; 6) Serious blood system, cardiovascular, liver, kidney disease or other disease affecting sexual activity; 7) Spinal cord injury; 8) Smoking, alcohol, drug abuse and masturbation history; 9) A history of drug abuse; (10) Receiving ED treatment or drugs that affect ED; Such as immunosuppressants, glucocorticoids, diuretics, receptor blockers, antioxidants, etc.; 11) History of mental illness, and the ED caused by anxiety, depression and other psychological factors was excluded according to the SAS standard score <50 and SDS standard score <53.

According to the above criteria, a total of 20 male T2DM patients were included and divided into DMED group (10 cases) and DM group (10 cases). The study was conducted in accordance with the Declaration of Helsinki,and with approval from The first hospital of Qiqihar ethics committee for clinical trials (2020-KY-007–01).Written informed consent was obtained from all the participants.

### Clinical Data of Patients

General information such as name, age, sex, height, weight, marital history, personal history, infection history, surgery and trauma history were collected. IIEF-5 scores, SAS scale and SDS scale were obtained using a questionnaire. Body mass index (BMI) was calculated by formula: BMI = Body weight/height^2^ (kg/m^2^). The remaining indicators were determined by clinical tests.

### Collection of Serum Samples

Clinical serum samples were collected by utilizing residual specimens from patients undergoing routine medical care. Each blood sample is 4–6 ml. Samples were then centrifuged and the supernatant was stored at −80°C in the centrifuge tube.

### Sample Sequencing

MiRNA sequencing was performed on a total of 20 cases, 10 cases in each group with no statistical difference in age, course of disease and BMI.

#### The Method of Sample Detecting

Use Agilent 2100 Bioanalyzer to test sample integrity and concentration, and NanoDrop to Inorganic ions orpolycarbonate contamination. This step aimed to provide a reference for library construction and later analysis.

#### Library Construction

Filter Small RNA: Use the 200ng-1 μg of RNA sample, then separate RNA segment of different size by PAGE gel, select 18–30 nt (14–30 ssRNA Ladder Marker, TAKARA) stripe and recycle; Adaptor ligation: Prepare connection 3′adaptor system (Reaction condition:70°C for 2min; 25°C for 2 h); Secondly add RT-Primer, (Reaction condition: 65°C for 15 min; ramp to 4°C at a rate of 0.3°C/s); Thirdly add 5′adaptor mix system (Reaction condition: 70°C for 2 min; 25°C for 1 h).

RT PCR: Prepare First Strand Master Mix and Super Script II (Invitrogen) reverse transcription (Reaction condition: 42°C for 1 h; 70°C for 15 min); Several rounds of PCR amplification with PCR Primer Cocktail and PCR Master Mix were performed to enrich the cDNA fragments (Reaction condition: 95°C for 3 min; 15–18 cycles of (98°C for 20 s, 56°C for 15 s, 72°C for 15 s); 72°C for 10 min; 4°C hold); Purify PCR products: Then the PCR products were purified with PAGE gel, dissolve the recycled products in EB solution.

#### Circularization

The double stranded PCR products were heat denatured and circularized by the splint oligo sequence. The single strand circle DNA (ssCir DNA) were formatted as the final library.

#### Library Quality Control

Library was validating on the Agilent Technologies 2100 bioanalyzer.

#### Sequencing

The library was amplified with phi29 to make DNA nanoball (DNB) which have more than 300 copies of one molecular. The DNBs were loaded into the patterned nanoarray and single end 50 bases reads were generated in the way of combinatorial Probe-Anchor Synthesis (cPAS).

### Feature Selection of Diabetic Mellitus Erectile Dysfunction Based on Support Vector Machine-Recursive Feature Elimination

Support Vector Machine-Recursive Feature Elimination (SVM-RFE) is a sequence backward selection algorithm based on the maximum interval principle of Support Vector Machine (SVM) (Guyon et al., 2002; Tang et al., 2018; Cheng et al., 2019; Yang et al., 2020; Liu et al., 2021b; Joshi et al., 2021).

The counts data based on miRNA sequencing were combined with the improved SVM-REF method proposed by Kai-Bo Duan et al. (Duan et al., 2005) to select miRNAs. Due to the randomness in Kai-Bo Duan’s method, in order to obtain a model with relatively small error, the whole process was repeated for 1,000 times. The model with the smallest error was selected as the final model. If there were multiple models with the same minimum error, the model with a large number of miRNAs was selected. The characteristic miRNAs, called DMEDSig-4, were finally screened.

### Differentially Expressed

According to the identified DMEDSig-4, we combined the miRNA read counts data calculated by the sequencing process. In view of the negative binomial distribution of counts data, we used the R package DESeq2 to calculate the differences between the DM groups and DEMD groups (Love et al., 2014).

### Identification of miRNA Target Genes

The miRNA-targeted mRNAs of DMEDSig-4 were pooled using the online bioinformatics analysis tool (EncoRI). Firstly, we searched EncoRI database (http://starbase.sysu.edu.cn/) (Li et al., 2014), which included seven databases (microT, miRanda, miRmap, PITA, RNA22, PicTar and TargetScan). Then, we entered characteristic miRNA of DMEDSig-4, set CLIP-Data≥5, Program-Number≥5, and Degradome-Data≥1 to obtain miRNA target genes.

### Analysis of Gene Function Enrichment Regulatory Network

The R package FGNet allows functional enrichment analysis (FEA) to be performed on a list of genes or expression sets and the results to be converted into a network (Aibar et al., 2015; Azimi et al., 2021). The network can provide an overview of the biological function of genes/terms, and allows easy seeing of links between genes, the overlap between clusters, etc. We selected the annotation tool topGO for functional annotation of target genes. GO was used to describe gene functions along with three aspects: biological process (BP), cellular component (CC) and molecular function (MF). The *p* < 0.01 was considered significant.

## Results

### Statistical Analysis of Clinical Data

Firstly, we collected 20 high-quality samples (10 DMED and 10 DM) from 60 patients according to the inclusion criteria. Then, we collected and collated the clinical information of these 20 samples. Subsequently, statistical analysis was performed for the DMED group and the DM group, including age, diabetes duration, BMI, fasting plasma glucose, glycated hemoglobin, total cholesterol, triglyceride, testosterone, thyroid stimulating hormone, serum creatinine, carbamide, alanine aminotransferase, aspartate aminotransferase. Meanwhile, we also conducted a questionnaire survey on these 20 patients, and obtained IIEF-5 score, self-rating anxiety scale (SAS) score and self-rating depression scale (SDS) score. In this project, a total of 20 samples were tested using DNBSEQ platform. The average ratio of sample to genome was 78.22%. A total of 1,044 small RNAs were detected.

SAS9.4 international standard statistical programming software was used for statistical analysis. Measurement data processing normal distribution and variance homogeneity were measured, The measurement data conforming to normal distribution are expressed by mean ± standard deviation,comparison between two groups with sample t tests,non normal distribution adopt median and IQR to express, makes the non-parametric test. *p* < 0.05 could be considered statistically significant (Table 1). Finally, according to the results, there were no statistically significant differences with regard to age, diabetes duration and BMI (*p* > 0.05). In addition, there was no significant difference in other statistical indicators (*p* > 0.05) except IIEF-5 (*p* < 0.05). This showed that we chose samples to avoid the influence of other factors as much as possible.

**TABLE 1:** Comparisons of clinical data. BMI, body mass index; IIEF-5, international index of erectile function 5; SAS, self-rating anxiety scale; SDS, self-rating depression scale; FPG, fasting plasma glucose; HbA1c, glycated hemoglobin; TC, total cholesterol; TG, triglyceride; TT, testosterone; TSH, thyroid stimulating hormone; Scr, serum creatinine; Urea, carbamide; ALT, alanine aminotransferase; AST, aspartate aminotransferase.

Parameter	Group (DMED)	Group (DM)	*p* Value
Age, years	48.7 ± 5.03	46.6 ± 6.19	0.416
Diabetes duration, years	4 (2–8)	2 (2–5)	0.147
BMI, kg/m^2^	26.19 ± 3.08	26.71 ± 3.39	0.725
IIEF-5 score	12.30 ± 4.85	23.40 ± 1.07	<0.0001***
SAS score	41.7 ± 4.9	40 ± 6.5	0.517
SDS score	48.5 (46–51)	50 (50–51)	0.244
FPG, mmol/l	12.61 (8.29–14.11)	9.46 (8.31–10.02)	0.364
HbA1c, %	9.75 ± 2.63	8.56 ± 1.59	0.236
TC, mmol/l	5.54 ± 1.15	4.87 ± 1.33	0.243
TG, mmol/l	2.4 ± 1.41	2.99 ± 2.09	0.468
TT, ng/ml	4.08 ± 1.11	3.87 ± 1.01	0.658
TSH, uIU/ml	2.21 ± 0.69	1.75 ± 0.95	0.225
Scr, umol/L	70.8 ± 11.03	63 ± 6.25	0.067
UREA, mmol/L	5.88 ± 1.24	6.03 ± 1.43	0.813
ALT	18.8 (16.5–21.4)	25.15 (19.8–34.5)	0.059
AST	16.18 ± 3.92	21 ± 8.93	0.136

### Construction of microRNAs Signature Associated With Diabetic Mellitus Erectile Dysfunction

Many pieces of evidence have shown that miRNAs have diagnostic and predictive value for DMED in mice (Li et al., 2017; Cong et al., 2020; Huo et al., 2020). However, few correlational researches of DMED have been conducted on the human body. In order to further explore the mechanism of miRNAs regulating DMED in the human body, we selected 10 DM groups and 10 DMED groups for miRNA sequencing based on the inclusion criteria. The miRNA counts data of 20 samples were obtained. Firstly, miRNA counts data were filtered to delete miRNAs with counts of 0 in most samples. The selected miRNAs should have counts of non-0 in at least 18 samples. Due to the limited sample size of miRNA expression profiles in DM groups and DMED groups, we adopted the machine learning method of SVM-RFE to screen characteristic miRNAs (Sanz et al., 2018).

Firstly, the miRNA counts data contained all miRNAs that will be imported. Secondly, the algorithm used SVM model training samples to calculate the weight of each miRNA. Subsequently, we ranked miRNAs according to their weights and deleted the bottom-ranked miRNAs from the subset (Lin et al., 2017). Meanwhile, the remaining miRNAs were used to train the model again for the next iteration. Finally, the required number of miRNAs were selected. The later the miRNA was removed from the subset, the more significant the miRNAs were. We employed SVM-RFE machine learning method. Firstly, SVM-RFE method can be used for linear/nonlinear classification as well as regression with low generalization error rate. That is to say, he has a good learning ability, and the results of learning have a good extension. Secondly, SVM-RFE method can solve the problem of machine learning in the case of small samples, solve the problem of high dimension, and avoid the problem of neural network structure selection and local minimum point. Finally, SVM-RFE method is the best off-the-shelf classifier and can get a low error rate. Meanwhile, SVM-RFE method can make good classification decisions for data points outside the training set.

**FIGURE 1:**
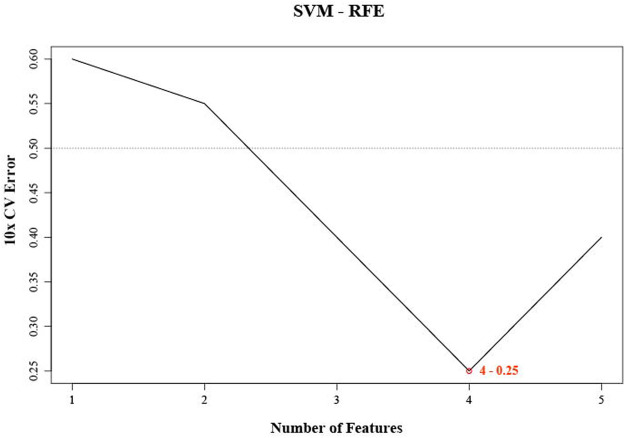
SVM-RFE were used for feature selection. The point highlighted indicates the lowest error rate, and the corresponding miRNA at this point are the best signature selected by SVM-RFE.

Since SVM-REF was more sensitive to feature changes, the ranking of features was different each time. For the robustness of feature selection, we refer to the method of Kai-Bo Duan (Duan et al., 2005). We used ten-fold cross-validation here by adding resampling to each iteration to stabilize the ranking (Zhu et al., 2021b). After 1,000 cycles of the algorithm (Supplementary Table S1), four characteristic miRNAs (hsa-let-7E-5p, hsa-miR-30 days-5p, hsa-miR-199b-5p, and hsa-miR-342–3p) were obtained according to the lowest error rate of 0.25 in ten-fold cross-validation (Figure 1). We found that the error rate decreased significantly from the first to the fourth feature number, and then increased significantly from the fifth. Obviously, the feature number of four had the best differentiation between DMED groups and DM groups. These four miRNAs (hsa-let-7E-5p, hsa-miR-30 days-5p, hsa-miR-199b-5p, and hsa-miR-342–3p) obtained by machine learning methods had the best classification performance in the DMED and DM groups. Therefore, we referred to this predictive signature as DMEDSig-4. In the following results, the performance of DMEDSig-4 was verified and analyzed.

### Classification Capability Verification and Clinical Association Analysis of Diabetic Mellitus Erectile DysfunctionSig-4

**FIGURE 2:**
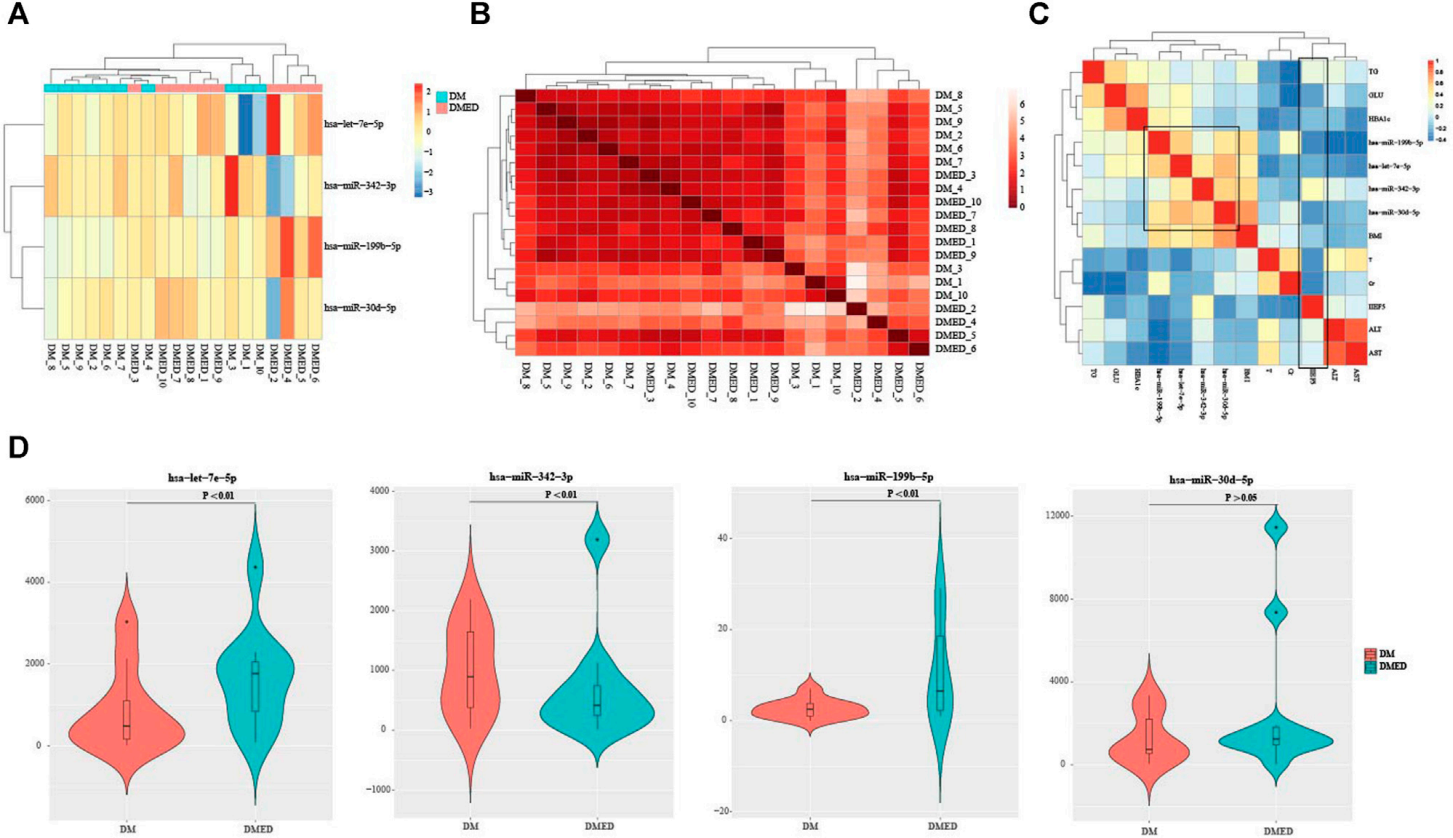
The verification of DMEDSig-4 classification ability and the association of clinical information. **(A)** Clustering heat map of DMEDSig-4 in DM and DMED groups. Red: Up-regulated expression; Blue: Down-regulated expression. **(B)** Heat map of similarity between DM groups and DMED groups. **(C)** Correlation analysis of DMEDSig-4 and clinical factors. BMI, body mass index, IIEF5, international index of erectile function 5; HbA1c, glycated hemoglobin; TT, testosterone; GLU, glucose; Cr, creatinine; TG, triglyceride; ALT, Alanine transaminase; AST, aspartic acid transaminase. **(D)** Violin diagram of differential expression of DMEDSig-4 in DM and DMED samples.

In order to investigate the expression patterns of four characteristic miRNAs predicted by DMEDSig-4, we first conducted hierarchical clustering according to counts data of these four miRNAs and plotted the clustering heat map of DMEDSig-4 in 20 samples (Figure 2A). The results of hierarchical clustering analysis showed that the 20 samples were clustered into three clusters by DMEDSig-4. The two clusters on the right were completely composed of samples from the DM or DMED groups. Although the cluster on the left was a mixture of the two types of samples, except for DMED_3, DM and DMED were significantly clustered into the same cluster. This phenomenon indicated that all samples were characterized by the DMEDSig-4 expression pattern. In addition, we also calculated the similarity between samples according to Euclidean distance (Figure 2B). The results showed that the Euclidean distance between some samples was very small, indicating these samples had a high similarity. For example, samples DM_8, DM_5, DM_9, DM_2, DM_6 and DM_7. Samples DMED_10, DMED_7, DMED_8, DMED_1 and DMED_9.

In order to further explore the correlation between DMEDSig-4 and clinical factors, we calculated the Spearman correlation between miRNAs and clinical indicators of all samples (Figure 2C, Supplementary Table S2). It could be observed that the four miRNAs in DMEDSig-4 were positively correlated with each other, indicating that there might be a mechanism of co-operative regulation of DMEDSig-4. In general, the diagnosis of ED depends on the history of disease and the IIEF-5 (Rosen et al., 1997; Hatzichristou et al., 2002). It could be observed that IIEF-5 was negatively correlated with DMEDSig-4. Meanwhile, IIEF-5 was negatively correlated with testosterone, glycated globin, creatinine, fasting blood glucose and other indicators. Previous studies had verified that testosterone was a protective factor in DMED (Diaz-Arjonilla et al., 2009), and the negative correlation between testosterone and indicators confirmed the correctness of IIEF-5 data.

In order to verify the classification ability of the four characteristic miRNAs in DMEDSig-4 for samples, we calculated the significant difference of miRNA expression between the DM groups and the DMED groups. The results showed that the differences of hsa-let-7e-5p, hsa-miR-199b-5p and hsa-miR-342–3p were significant (*p* < 0.01), while the effect of hsa-miR-30 days-5p was not significant (*p* > 0.05) (Figure 2D).

These results indicated that the miRNAs we identified have certain characteristics between the DM and DMED groups, regardless of the individual miRNA level or the overall expression pattern, and there were also certain correlations with clinical indicators. These showed that DMEDSig-4 had a certain role in helping to identify DMED.

### Identification of Target microRNAs of Diabetic Mellitus Erectile DysfunctionSig-4

**FIGURE 3:**
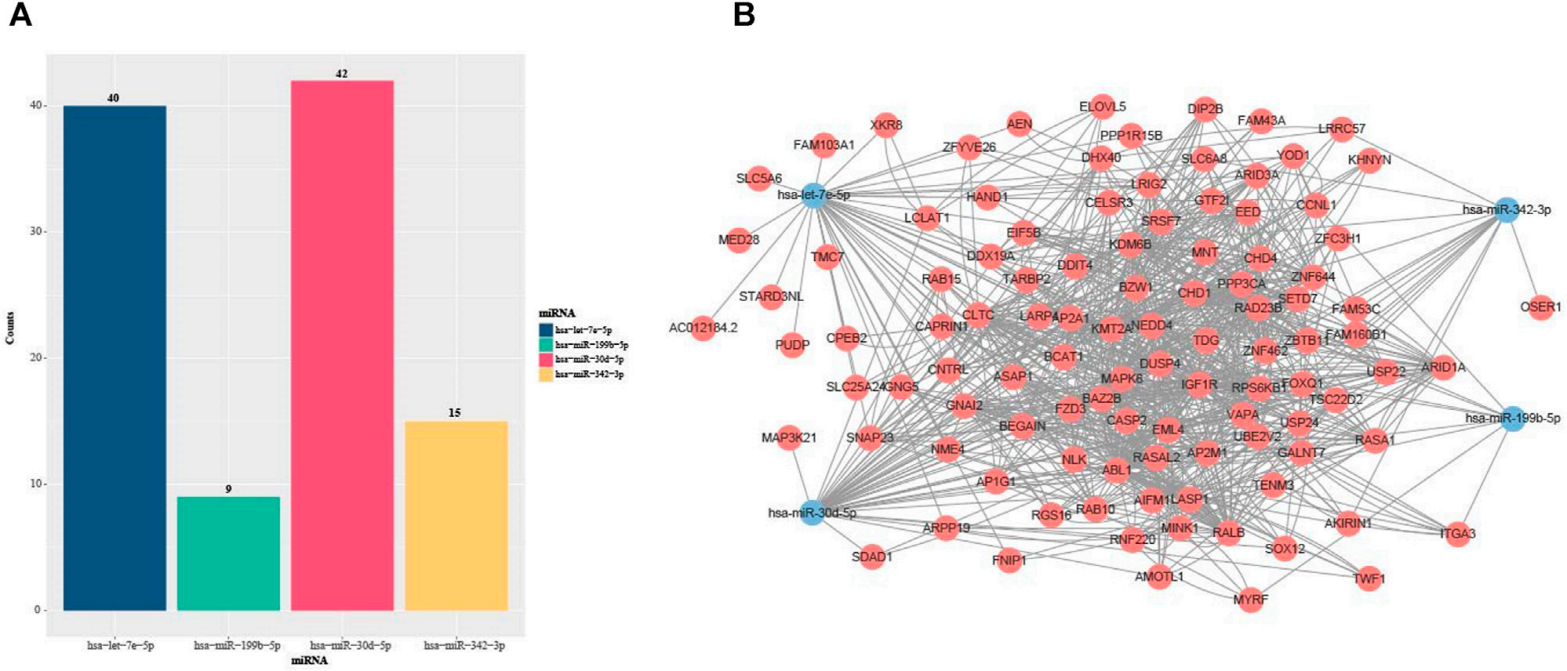
Statistics of miRNA target miRNAs in DMEDSig-4. **(A)** Bar chart of DEMDSig-4 target genes **(B)** miRNA-mRNA interaction network diagram of DEMDSig-4. Red: target mRNAs; Blue: miRNAs of DMEDSig-4.

Many pieces of evidence demonstrated that miRNAs were capable of regulating various biological and pathological processes via inhibiting target mRNA translation or promoting mRNA degradation (Fabian et al., 2010; Fang et al., 2019; Riaz and Li, 2019; Wang et al., 2020). Meanwhile, the miRNAs could act as signatures of disease, strong indicators of prognosis or potential therapeutic targets (Zhu et al., 2011; Huang, 2018; Norsworthy et al., 2020). In order to further study the effects of the DMEDSig-4 target mRNAs on ED, we used the online bioinformatics database ENCORI (http://starbase.sysu.edu.cn/) to identify the target mRNAs. According to the parameters set, the target genes corresponding to each miRNA were obtained. The results of predicting targeted mRNAs on four miRNAs showed that there were 40 targeted mRNAs for hsa-let-7e-5p, 42 targeted mRNAs for hsa-miR-30 days-5p, nine targeted mRNAs for hsa-miR-199b-5p, and 15 targeted mRNAs hsa-miR-342–3p (Figure 3A).

In order to observe the relationship between miRNAs and target mRNAs, we constructed the miRNA-mRNA interaction network diagram based on DMEDSig-4 and target mRNAs through STRING database (https://string-db.org/) (Figure 3B). The network consisted of four miRNA nodes, 105 mRNA nodes and 870 edges. Among them, 764 edges were the relationship between target mRNAs, and 106 edges were the relationship between miRNA and target mRNAs. There was only one intersection among miRNA target mRNAs, indicating that these miRNAs did not tend to jointly regulate a target mRNA, and the identified target mRNAs had a close interaction relationship, suggesting that these mRNAs might act together on the same pathway.

### Functional Annotation of microRNAs and Its Target microRNAs

**FIGURE 4:**
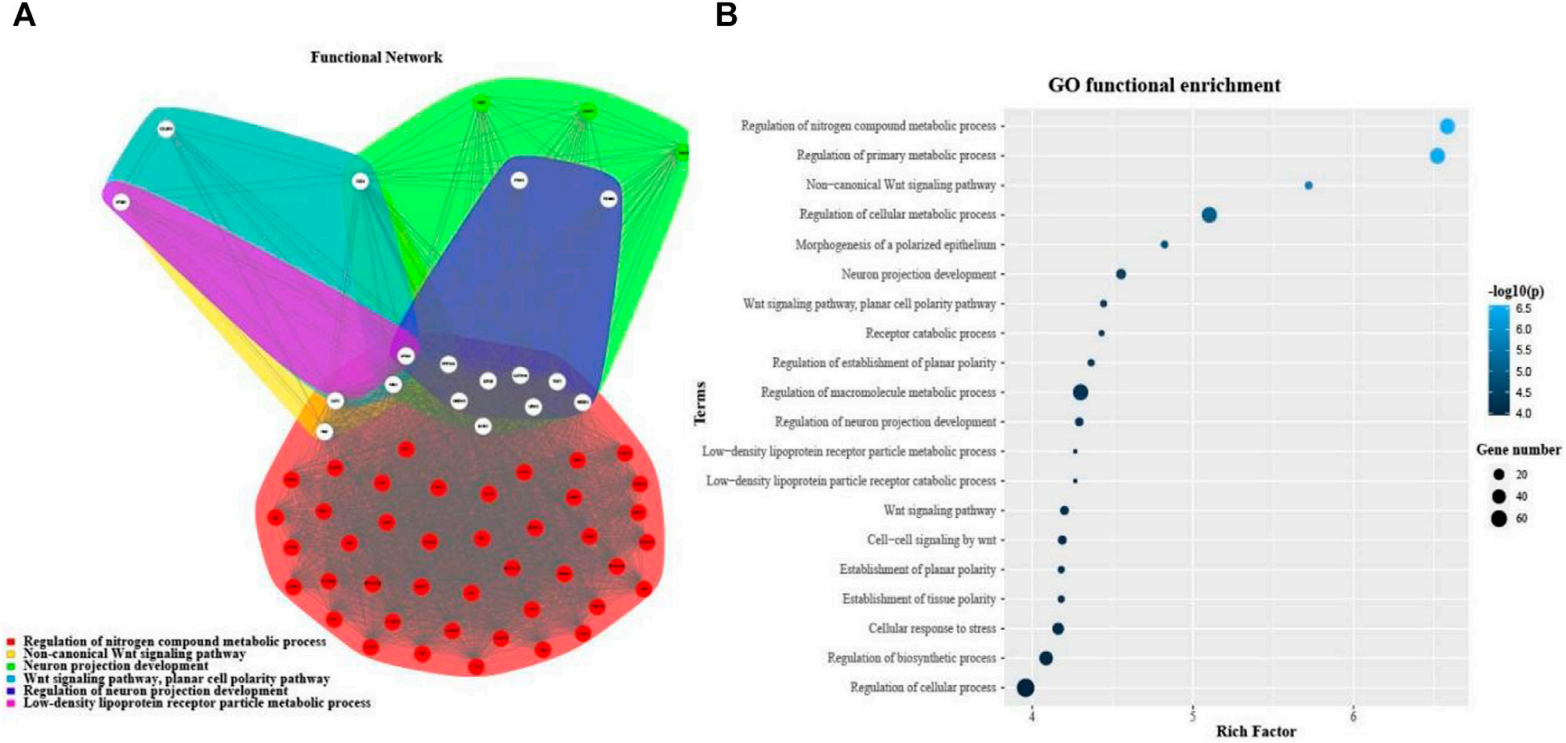
Functional annotation of DMEDSig-4. **(A)** Functional Gene Network (FGN) of clusters defined by topGO for DMEDSig-4 Targeted Genes. Edges: link genes annotated under the same enrichment terms. Node colors: functional clusters containing genes. White nodes: hub genes expressed in multiple clusters. **(B)** Bubble chart of function enrichment results of DMEDSig-4.

In order to investigate the function of DEMDSig-4 targeted mRNAs, we used the topGO (Yang et al., 2021) annotation tool in R package FGNet for functional annotation of DEMDSig-4. We aimed to the functional analysis of each target mRNA searching further to verify the characteristic function of DEMDSig-4. The resulting network represented the links and associations between clusters of mRNAs and enriched terms. We annotated the biological process (BP), cellular component (CC) and molecular function (MF) of the target mRNAs. A total of 255 clusters and descriptions, we provided in the form of supplementary files (Supplementary Table S3). Here, we focused on the biological process of the target mRNAs. Due to the large number of biological processes and the complexity of the network, we manually selected representative biological processes for demonstration, including most of the mRNAs targeted for DMEDSig-4 (Figure 4A). The biological process includes: Regulation of nitrogen metabolic Process non-canonical Wnt signaling Pathway Neuron Projection Development “Wnt Signaling Pathway, Planar Cell Polarity Pathway” Regulation of Neuron projection Development, And “Density Lipoprotein receptor particle metabolic Process”. We found that the subnetwork was divided into two broad functional categories, including metabolic function and neural function, which were closely related to the pathological mechanism of ED (Müller and Mulhall, 2006; Shamloul and Ghanem, 2013).

Although FGNet provided a broad overview of the biological effects of human-specific genetic alterations by clustering functional terms within clusters and establishing relationships between such clusters, it lacked the detail that could be obtained by analyzing each functional category individually (Bitar et al., 2019; Zhang et al., 2021). Therefore, we turned to gene ontology (GO) analysis. The top 20 salient biological processes were functionally annotated in terms of *p* values (Figure 4B). The results demonstrated most of the signaling pathways were associated with DMED at the molecular and cellular levels, which could provide important information for revealing the most significant biological functions of DMED. We found that the biological processes of DMEDSig-4 were mainly divided into cell metabolism, neural signal transmission and planar cell polarity pathway. For example, we queried that the biological process “regulation of nitrogen Compound metabolic process” was associated with endothelial dysfunction (Andersson, 2003; Yuyun et al., 2018; Cyr et al., 2020). Endothelial dysfunction was recognized as a mainstay in the pathophysiology of the disease (Castela and Costa, 2016). As for the “WNT signaling pathway”, studies had demonstrated that the Wnt family contributed to the pathogenesis of diabetes-induced erectile dysfunction (Shin et al., 2014; Ghatak et al., 2017). ED was also involved in the regulation of the metabolic and nervous systems (Burnett, 2005; Ryan and Gajraj, 2012; Mitidieri et al., 2020). For example, GO terms included “regulation of primary metabolic process” “regulation of cellular metabolic process” and “cellular response to stress”. Literature verification showed that the above pathways were related to the pathological mechanism of DMED (Matfin et al., 2005; Zsoldos et al., 2019).

Finally, we annotated the characteristic miRNAs in DMEDSig-4 to prove the mechanism relationship between DMEDSig-4 and DMED. The expression of hsa-miR-342–3p helped to identify patients with cardiovascular disease (Seleem et al., 2019; Ray et al., 2020). Meanwhile, the expression level of this miRNA was significantly increased in diabetic nephropathy (Eissa et al., 2016; Jiang et al., 2020). Importantly, hsa-miR-342–3p was differentially expressed in obese children with and without endothelial dysfunction (Khalyfa et al., 2016), which was one of the important factors causing DMED. Meanwhile, hsa-miR-199b-5p, hsa-miR-30 days-5p and hsa-let-7e-5p were all related to diabetic kidney damage and cardiovascular diseases (Jia et al., 2016; Fedorko et al., 2017; Sun et al., 2018), which were all risk factors for ED.

## Discussion

Erectile dysfunction (ED) is a common and often overlooked complication of diabetes that can wreak havoc on men both physically and mentally. Studies have shown that type 2 diabetes mellitus (T2DM) is widely associated with ED and is a risk factor for ED. Interestingly, several studies have demonstrated that miRNAs are involved in the pathogenesis of ED. For example, Rama Natarajan et al. explored the role of miRNAs in the pathology of diabetic complications and also discussed the potential use of miRNAs as novel diagnostic and therapeutic targets for diabetic complications (Natarajan et al., 2012; Wang et al., 2019). Wang et al. found that upregulation of miR-320 was associated with impaired angiogenesis in diabetes (Wang et al., 2009). Yan Wen et al. found that miR-205 may contribute to the pathogenesis of DMED via down-regulation of androgen receptor expressions (Wen et al., 2019). Although a large number of studies have proved the regulatory relationship of miRNAs on ED, there is still a lack of research on the relationship between miRNAs and ED in the context of T2DM. This study aimed to further explore ED signature associated with diabetes by analyzing miRNA expression data in patients with DM. This signature may play a certain role in the diagnosis and treatment of DMED. This study is not only a preliminary attempt on miRNA signature of DMED, but also may serve as the basis for subsequent studies.

In terms of data, we collected a large amount of clinical information and conducted preliminary screening to select patients (disease history/clinical information) as similar as possible and to eliminate the interference of other factors to the greatest extent possible. Finally, we selected 10 DM patients and 10 DMED patients as the final study subjects. However, due to the limited time and cost, our patient cohort is still relatively small, and there is no additional data set verification, so the identified DMEDSig-4 may not have good universality, which will be our further research direction. In this study, we used a machine learning approach (SVM-RFE) to identify potential miRNA features in a sample of DM and DMED patients. First, four optimal feature miRNAs (hsa-miR-342–3p, hsa-miR-199b-5p, hsa-miR-30 days-5p and hsa-let-7e-5p) were identified after 1,000 cycles of the algorithm, called DMEDSig-4. They all had a good classification effect, and there might be a potential mechanism of co-regulation. Subsequently, after associating with clinical factors, we found that DMEDSig-4 was positively correlated with each other and negatively correlated with IIEF-5. Then, we searched for the miRNAs targeted by DMDESig-4 and constructed a miRNA-mRNA interaction network. The results showed that the network consisted of four miRNA nodes, 105 mRNA nodes and 870 edges. Meanwhile, there was only one intersection between the targeted miRNAs of miRNA, indicating that these miRNAs did not tend to jointly regulate a target mRNA. Importantly, the identified target mRNAs had a close interaction relationship, suggesting that these mRNAs might act together on the same pathway. This might play an enlightening role in the subsequent studies of miRNA on DMED. Finally, we searched for the miRNAs targeted by DMDESig-4 and performed functional enrichment. The results showed that the DMDESig-4 pathways were closely related to DM and ED, which might contribute to the pathogenesis of ED. The literature review has shown that DMEDSig-4 was associated with cardiovascular disease, diabetic nephropathy and liver injury, which were all potential risk factors for ED (Hu et al., 2021). Clinical ED patients are often accompanied by cardiovascular disease, kidney and liver damage and other symptoms.

We hope that the characterization of miRNAs will contribute to a comprehensive understanding of their pathways in DMED and improve therapeutic strategies for patients with DMED. We hope that the identification of DMEDSig-4 will contribute to a comprehensive understanding of its pathway mechanism in DMED and improve therapeutic strategies for patients with DMED.

## Data Availability

The datasets presented in this study can be found in online repositories. The names of the repository/repositories and accession number(s) can be found below: https://www.ncbi.nlm.nih.gov/geo/query/acc.cgi?acc=GSE182053.
